# A novel image integration technology mapping system significantly reduces radiation exposure during ablation for a wide spectrum of tachyarrhythmias in children

**DOI:** 10.3389/fped.2023.1148745

**Published:** 2023-04-06

**Authors:** Jiang He, Zhang Yi, Li Meiting, Zhou Huiming, Li Jinhao, Chen Danlei, Li Xiaomei

**Affiliations:** ^1^ Department of Cardiology, Children's Hospital, Capital Institute of Pediatrics, Beijing, China; ^2^ Department of Pediatrics, Heart Center, The First Affiliated Hospital of Tsinghua University, Beijing, China

**Keywords:** child, fluoroscopy, cardiac arrhythmias, ablation, 3D mapping

## Abstract

**Objective:**

Radiofrequency catheter ablation (RFCA) has evolved into an effective and safe technique for the treatment of tachyarrhythmia in children. Concerns about children and involved medical staff being exposed to radiation during the procedure should not be ignored. “Fluoroscopy integrated 3D mapping”, a new 3D non-fluoroscopic navigation system software (CARTO Univu Module) could reduce fluoroscopy during the procedure. However, there are few studies about the use of this new technology on children. In the present study, we analyzed the impact of the CARTO Univu on procedural safety and fluoroscopy in a wide spectrum of tachyarrhythmias as compared with CARTO3 alone.

**Methods:**

The data of children with tachyarrhythmias who underwent RFCA from June 2018 to December 2021 were collected. The CARTO Univu was used for mapping and ablation in 200 cases (C3U group) [boys/girls (105/95), mean age (6.8 ± 3.7 years), mean body weight (29.4 ± 7.9 kg)], and the CARTO3 was used in 200 cases as the control group (C3 group) [male/female (103/97), mean age (7.2 ± 3.9 years), mean body weight (32.3 ± 19.0 kg)]. The arrhythmias were atrioventricular reentrant tachycardia (AVRT, *n* = 78), atrioventricular node reentrant tachycardia (AVNRT, *n* = 35), typical atrial flutter (AFL, *n* = 12), atrial tachycardia (AT, *n* = 20) and ventricular arrhythmias [VAs, premature ventricular complexes or ventricular tachycardia, *n* = 55].

**Results:**

① There was no significant difference in the acute success rate, recurrence rate, and complication rate between the C3 and C3U groups [(94.5% vs. 95.0%); (6.3% vs. 5.3%); and (2.0% vs. 1.5%); *P *> 0.05]. ② The CARTO Univu reduced radiation exposure: fluoroscopy time: AVRT C3: 8.5 ± 7.2 min vs. C3U: 4.5 ± 2.9 min, *P *< 0.05; AVNRT C3: 10.7 ± 3.2 min vs. C3U: 4.3 ± 2.6 min, *P *< 0.05; AT C3: 15.7 ± 8.2 min vs. C3U: 4.5 ± 1.7 min, *P *< 0.05; AFL C3: 8.7 ± 3.2 min vs. C3U: 3.7 ± 2.7 min, *P *< 0.05; VAs C3: 7.7 ± 4.2 min vs. C3U: 3.9 ± 2.3 min, *P *< 0.05. Corresponding to the fluoroscopy time, the fluoroscopy dose was also reduced significantly. ③ In the C3U group, the fluoroscopy during VAs ablation was lower than that of other arrhythmias (*P *< 0.05).

**Conclusion:**

The usage of the “novel image integration technology” CARTO Univu might be safe and effective in RFCA for a wide spectrum of tachyarrhythmias in children, which could significantly reduce fluoroscopy and has a more prominent advantage for VAs ablation.

## Introduction

Radiofrequency catheter ablation (RFCA) has become the main treatment for tachyarrhythmias in children in the past ten years (1, 2). Concerns about children and involved medical staff being exposed to radiation during the procedure should not be ignored. Some new technologies for non-fluoroscopic visualization of the catheter are desirable. “Fluoroscopy integrated 3D mapping”, a new 3D non-fluoroscopic navigation system software (CARTO Univu Module of CARTO3 system, Biosense Webster), was developed and allows electro-anatomical localization of diagnostic and ablation catheters in pre-recorded x-ray images or non-gated x-ray videos. It could reduce fluoroscopy during the procedure. The application of CARTO Univu has been shown to further reduce fluoroscopy in adults (3), however, there are few studies about the use of this new technology on children. This study aimed to evaluate the impact of the CARTO Univu module on procedural safety and fluoroscopy in a wide spectrum of cardiac arrhythmias as compared to the use of the 3D-mapping system CARTO3 (Biosense Webster) alone in children.

## Materials and methods

### Study population and procedures

The data of children undergoing RFCA for tachyarrhythmias, from June 2018 to December 2021, were collected, including atrioventricular reentry tachycardia (AVRT), atrioventricular nodal reentry tachycardia (AVNRT), atrial tachycardia (AT), atrial flutter (AF), and ventricular arrhythmias (VAs, including premature ventricular beats and ventricular tachycardia). Electrophysiological studies and RFCA in children are the same as the conventional way of our center (4). All procedures were indicated according to current guidelines.

In the present study, 200 cases were ablated using the CARTO Univu module (C3U) and 200 cases were ablated using the CARTO3 alone as the control group (C3) for comparison.

This study was a retrospective, single-center study, approved by the Medical Ethics Review Board of our hospital (2018-04).

### Technology description

In the C3U group, we began the CARTO3 system and then performed CARTO3 Univu registration.

After completing CARTO Univu registration, x-ray cardiac images were recorded in the right anterior oblique position 30° (RAO30°), left anterior oblique position 45° (LAO45°), or other special positions as needed. These prerecorded images could be inputted into CARTO3 system as the background during the procedure. Then, the electroanatomic catheter visualization was shown in these prerecorded x-ray images.

**Figure 1:**
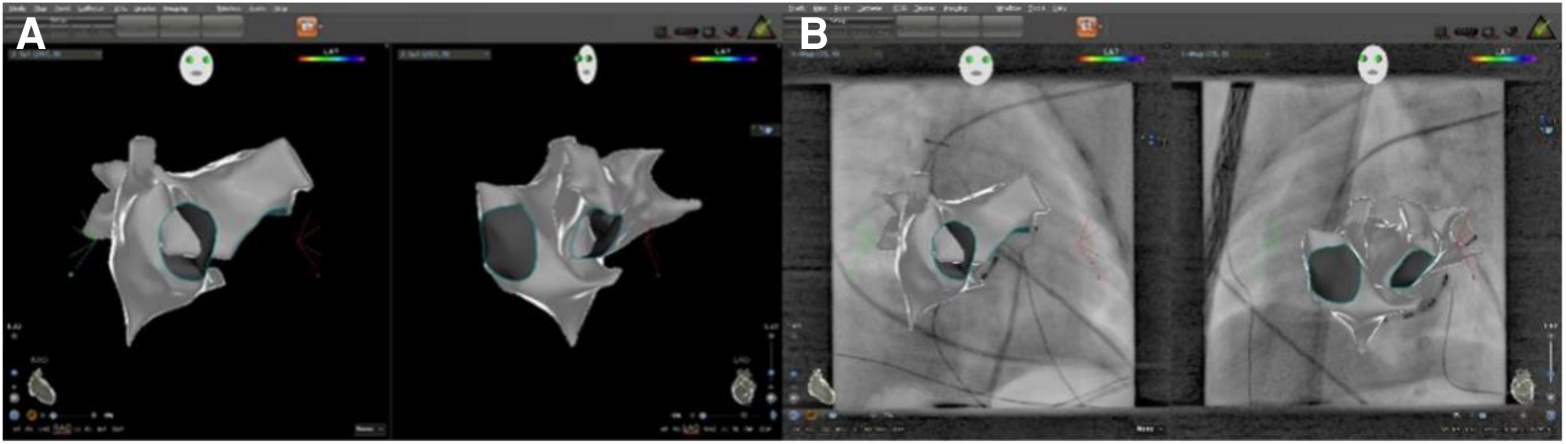
The cardiac 3D model images of CARTO3 and CARTO univu. Slide (**A**) shows the CARTO3 cardiac 3D model image with a right anterior oblique position of 30° and a left anterior oblique position of 45° on a black background; Slide (**B**) shows the CARTO Univu model, which puts the prerecorded x-ray cardiac image into the CARTO3 system and allows mapping and ablation on the background of x-ray cardiac images.

The model images of CARTO3 and CARTO Univu are shown in Figure 1.

### Follow-up

Follow-ups at 1 month, 3 months, and 6 months were performed after RFCA. Recurrence was defined as the presence of the same symptomatic tachycardia and preexcitation at follow-up.

### Statistical methods

SPSS 22.0 software was used to complete the statistical analysis of the study data. Continuous variables are expressed as mean ± SD and categorical variables as numbers and percentages. The *t*-test was used for continuous variables statistics and the chi-square test for categorical variables statistics. The difference was considered statistically significant with a two-sided *P *< 0.05.

## Results

### Characteristics of patients

C3U group: 105 boys, 95 girls, age (6.8 ± 3.7 years) (1.2–17 years), weight (29.4 ± 7.9 kg) (8.2–105 kg). C3 group: 103 boys, 97 girls, age (7.2 ± 3.9 years) (0.9–17 years), weight (32.3 ± 19.0 kg) (8.9–123 kg).

The types of tachyarrhythmias in children in both groups were: 78 cases of AVRT, 35 cases of AVNRT, 20 cases of AT, 12 cases of AF, and 55 cases of VAs.

### Comparison of ablation outcomes between the groups

There was no significant difference in the acute success rate, recurrence rate, and complication rate of ablation between the C3 and C3U groups [(94.5% vs. 95.0%); (6.3% vs. 5.3%); (2.0% vs.1.5%); *P *> 0.05].The mean procedure time was longer in the C3U group than in the C3 group (101.2 ± 37.3 min vs. 91.2 ± 41.2 min), with a statistically significant difference (*P* < 0.05). The details are shown in Table 1.

**Table 1:** Comparison of ablation outcomes between the C3 and C3U groups (*n* = 200).

Type (*n*)	Duration of procedure (min)			Success *n* (%)			Recurrence *n* (%)		Complications *n* (%)	
	C3	C3U	*P*	C3	C3U	*P*	C3	C3U	C3	C3U
AVRT (78)	91.8 ± 36.6	101.8 ± 43.6	0.11	73 (93.6)	75 (96.2)	0.46	5 (6.8)	5 (6.7)	3	3
AVNRT (35)	103.3 ± 26.9	113.7 ± 39.0	0.26	35 (100)	34 (97.1)	0.31	0 (0)	0 (0)	0	0
AT (20)	115.7 ± 59.7	126.3 ± 43.0	0.39	16 (80)	17 (85.0)	0.67	4 (25)	3 (17.6)	0	0
AF (12)	78.5 ± 43.7	81.6 ± 32.2	0.84	12 (100)	12 (100)	/	1 (8.3)	0 (0)	0	0
VA (55)	71.3 ± 39.0	82.8 ± 28.5	0.12	51 (92.7)	50 (90.9)	0.72	2 (3.9)	2 (4.0)	1	0
Total (200)	91.2 ± 41.2	101.2 ± 37.3	0.01	189 (94.5)	190 (95.0)	0.82	12 (6.3)	10 (5.3)	4 (2.0)	3 (1.5)

AVRT, atrioventricular fold tachycardia; AVNRT, atrioventricular nodal fold tachycardia; AT, atrial tachycardia; AF, atrial flutter; VAs, ventricular arrhythmias, including premature ventricular beats and ventricular tachycardia.

### Comparison of fluoroscopy between the groups

**Figure 2:**
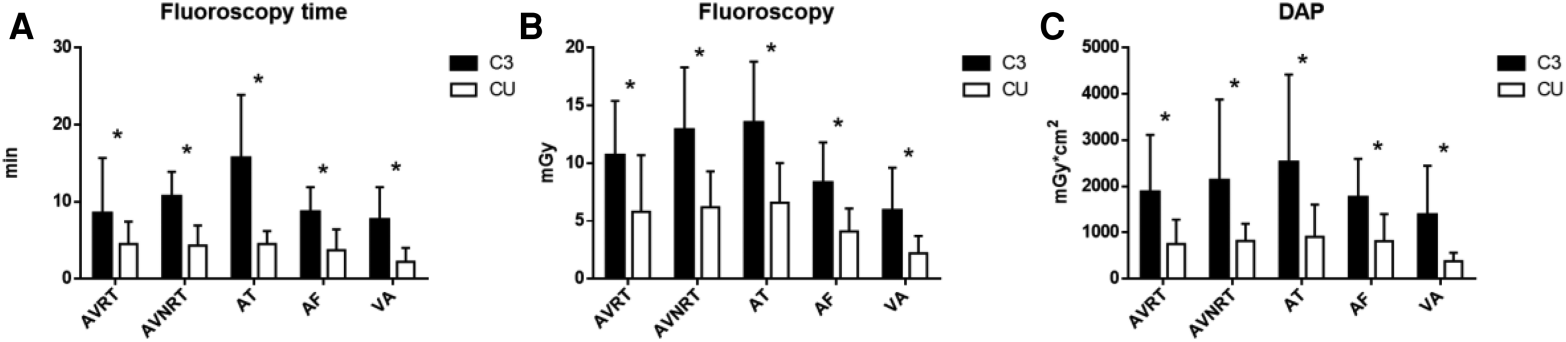
Comparison of fluoroscopy during procedures between the C3 and C3U groups. (**A**) Shows the fluoroscopy time; (**B**) shows the fluoroscopy dose; and (**C**) shows the dose area product (DAP).

There was a significant difference in fluoroscopy reduction in the C3U group compared with the C3 group during the procedure [fluoroscopy time: (3.9 ± 2.3) min vs. (9.9 ± 5.7) min; fluoroscopy dose (5.2 ± 2.8) mGy vs. (10.1 ± 4.7) mGy; DAP: (734.1 ± 514.4) mGy.cm^2^ vs. (1903.2 ± 1416.5) mGy.cm^2^; *P* < 0.05]. The details are shown in Table 2, Figure 2.

**Table 2:** Comparison of fluoroscopy between the C3 and C3U groups (*n* = 200).

	Fluoroscopy duration (min)			Fluoroscopy dose (mGy)			DAP (mGy.cm^2^)		
Type (*n*)	C3	C3U	*P*	C3	C3U	*P*	C3	C3U	*P*
AVRT (78)	8.5 ± 7.2	4.5 ± 2.9	<0.05	10.7 ± 4.7	5.8 ± 4.9	<0.05	1876.5 ± 1236.3	751.1 ± 528.8	<0.05
AVNRT (35)	10.7 ± 3.2	4.3 ± 2.6	<0.05	12.9 ± 5.4	6.2 ± 3.1	<0.05	2169.7 ± 1753.6	818.0 ± 370.2	<0.05
AT (20)	15.7 ± 8.2	4.5 ± 1.7	<0.05	13.5 ± 5.3	6.6 ± 3.4	<0.05	2524.1 ± 1896.4	908.2 ± 697.3	<0.05
AF (12)	8.7 ± 3.2	3.7 ± 2.7	<0.05	8.3 ± 3.5	4.1 ± 2.0	<0.05	1759.5 ± 838.9	812.6 ± 591.5	<0.05
VA (55)	7.7 ± 4.2	2.2 ± 1.8	<0.05	5.9 ± 3.7	2.2 ± 1.5	<0.05	1386.3 ± 1057.1	380.5 ± 184.4	<0.05
Total (200)	9.9 ± 5.7	3.9 ± 2.3	<0.05	10.1 ± 4.7	5.2 ± 2.8	<0.05	1903.2 ± 1416.5	734.1 ± 514.4	<0.05

AVRT, atrioventricular fold tachycardia; AVNRT, atrioventricular nodal fold tachycardia; AT, atrial tachycardia; AF, atrial flutter; VAs, ventricular arrhythmias, including premature ventricular beats and ventricular tachycardia; DAP, dose area product.

### Comparison of fluoroscopy for different types of tachyarrhythmias in the C3U group

**Figure 3:**
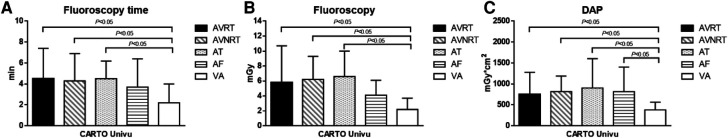
Comparison of fluoroscopy for different types of tachyarrhythmias in the C3U group. (**A**) Shows the fluoroscopy time; (**B**) Shows the fluoroscopy dose; (**C**) Shows the dose area product (DAP).

In the C3U group, the fluoroscopy in ablation for ventricular arrhythmias was lower than that of other arrhythmias (*P *< 0.05). The details for different types of tachyarrhythmias within the C3Un group were shown in Figure 3.

## Discussion

This study shows that CARTO Univu could significantly reduce fluoroscopy compared with CARTO3 alone during RFCA for tachyarrhythmias in children while ensuring the safety and efficacy of the procedure. In addition, it could reduce fluoroscopy more significantly in ventricular arrhythmias ablation than in other types of tachyarrhythmias.

Multicenter registry studies have shown that RFCA for children with tachyarrhythmias is safe and has a high success rate (5, 6). However, fluoroscopy during the procedure has some adverse effects on both patients and operators, such as skin damage, tumors, and genetic defects (7). Children have a longer life and a longer window of exposure to radiation than adults. In addition, children are more sensitive to fluoroscopy when they are growing and developing. Therefore, reducing fluoroscopy during the procedure is very important for children, and the clinical principle of ALARA (As Low As Reasonably Achievable) should be followed in children's ablation (8). In recent years, with the application of 3D electroanatomical mapping systems, it is possible to avoid the anatomical positioning of the catheter under x-ray fluoroscopy and significantly reduce the x-ray radiation (9). Miyake et al. applied the 3D electroanatomical mapping system to guide the ablation of supraventricular tachycardia in children, and the fluoroscopy dose was reduced from 387 mGy to 110 mGy, and the fluoroscopy time was reduced by 59% (18.3 min vs. 7.5 min) compared with the traditional mapping and ablation procedure *via* x-ray (10). Spar et al. also showed that by using a 3D mapping system to ablate children's tachycardias, the fluoroscopy time was significantly shorter (11). However, the 3D electroanatomical mapping system still has some limitations in children. Building the 3D cardiac model still needs to be done under x-ray fluoroscopy for safety reasons and with some children's arrhythmias and required complex mechanisms, the cumulative fluoroscopic time during the procedure is still long. In addition, some new technologies for non-fluoroscopic visualization of catheters, such as intra-cardiac ultrasound, are not suitable for RFCA in children because of their bigger size or higher price.

The new technology, CARTO Univu, could integrate the 3D cardiac model with the prerecorded x-ray images so that the model and images could be displayed simultaneously without additional fluoroscopy. In addition, the x-ray image of two projection positions is displayed simultaneously, which greatly improves the accuracy of the spatial position of the catheter. CARTO Univu significantly reduces fluoroscopy and improves procedure efficiency. A recent study by Christoph et al. found that the CARTO Univu module significantly reduced fluoroscopy time and did not affect procedural outcomes in adult patients, and showed a more pronounced reduction in fluoroscopy using CARTO Univu module during ablating complex arrhythmias in adult patients, such as atrial fibrillation and ventricular tachycardia (3). Similar to the results in adults, our study, including a wide spectrum of children's cardiac arrhythmias and with a large sample size, showed no difference in the acute success rate, recurrence rate, and complication rate in the C3U group compared with the C3 group. It suggests that using the CARTO Univu module, RFCA for tachyarrhythmias in children is safe and effective. Notably, the fluoroscopy was significantly lower compared with the CARTO3 system alone, especially for ablation of complex arrhythmias, such as ventricular arrhythmias in children.

## Conclusion

The CARTO Univu system, a new fluoroscopy integrated technology, is safe and effective for the ablation of tachyarrhythmias in children, and significantly reduces fluoroscopy during the procedure, especially for ventricular arrhythmias ablation.

## Data Availability

The original contributions presented in the study are included in the article/Supplementary Material, further inquiries can be directed to the corresponding author.
